# Squeezing for Life – Properties of Red Blood Cell Deformability

**DOI:** 10.3389/fphys.2018.00656

**Published:** 2018-06-01

**Authors:** Rick Huisjes, Anna Bogdanova, Wouter W. van Solinge, Raymond M. Schiffelers, Lars Kaestner, Richard van Wijk

**Affiliations:** ^1^Department of Clinical Chemistry and Haematology, University Medical Center Utrecht, Utrecht University, Utrecht, Netherlands; ^2^Red Blood Cell Research Group, Institute of Veterinary Physiology, Vetsuisse Faculty and the Zurich Center for Integrative Human Physiology (ZIHP), University of Zurich, Zürich, Switzerland; ^3^Theoretical Medicine and Biosciences, Saarland University, Saarbrücken, Germany; ^4^Experimental Physics, Saarland University, Saarbrücken, Germany

**Keywords:** deformability, vesiculation, sickle cell anemia, thalassemia, hereditary spherocytosis, enzymopathies, hydration, hemolysis

## Abstract

Deformability is an essential feature of blood cells (RBCs) that enables them to travel through even the smallest capillaries of the human body. Deformability is a function of (i) structural elements of cytoskeletal proteins, (ii) processes controlling intracellular ion and water handling and (iii) membrane surface-to-volume ratio. All these factors may be altered in various forms of hereditary hemolytic anemia, such as sickle cell disease, thalassemia, hereditary spherocytosis and hereditary xerocytosis. Although mutations are known as the primary causes of these congenital anemias, little is known about the resulting secondary processes that affect RBC deformability (such as secondary changes in RBC hydration, membrane protein phosphorylation, and RBC vesiculation). These secondary processes could, however, play an important role in the premature removal of the aberrant RBCs by the spleen. Altered RBC deformability could contribute to disease pathophysiology in various disorders of the RBC. Here we review the current knowledge on RBC deformability in different forms of hereditary hemolytic anemia and describe secondary mechanisms involved in RBC deformability.

## Introduction

The primary function of RBCs is to enable respiration in tissues by providing oxygen and removing carbon dioxide via gas exchange in the lungs. During a typical 120 days lifespan of a RBC, it circulates through arteries, veins and small capillaries traveling –in total– a distance of 500 km (Lasch et al., 2000). RBC deformability, i.e., the ability of the RBC to change shape is essential for successful passage through these capillaries and splenic sinuses (Danielczok et al., 2017).

Deformability of RBC depends on the (i) structural properties of the “horizontal” cytoskeletal components such as spectrin (Burton and Bruce, 2011; Nans et al., 2011), (ii) vertical interaction of cytoskeleton and integral transmembrane complexes that is accomplished by ankyrin, 4.1 and 4.2 protein and the cytosolic domain of band 3 protein (also known as the anion exchanger 1 (AE1) or solute carrier family 4 member 1 (SLC4A1)) (Gallagher, 2004a) and (iii) resistance of the cytosolic pool (i.e., intracellular viscosity, RBC hydration state and surface-volume interaction) (Clark et al., 1983).

Deformability is also affected by metabolic processes controlling ATP levels and redox state. These factors control ion handling by pumps and passive transport pathways (Chu et al., 2012; Bogdanova et al., 2016), proteolytic activity of Ca^2+^-dependent protease calpain (Bogdanova et al., 2013), and mutations and structural integrity of each element of the membrane architecture (Gallagher, 2004b). Failure to sustain deformability results in shortening of RBC life span and, when not compensated by *de novo* RBC production, in hemolytic anemia.

Therefore, reliable estimation of RBC deformability and understanding of the processes in control of it are essential for evaluation of severity of patients’ state and choosing of the optimal therapeutic strategy. This particularly relates to the feasibility of splenectomy as an option to improve or worsen condition of patients with anemic state (Iolascon et al., 2017).

In this review, we provide an overview of the current knowledge on the primary and secondary mechanisms involved in regulation of RBC deformability in hereditary hemolytic anemia. We discuss methodologies that are currently used to assess RBC deformability in the clinical and research laboratories. We link different processes, such as ion channel activity, intracellular energy metabolism and phosphorylation of membrane proteins to RBC deformability and illustrate how these processes are affected in various RBC pathologies, such as sickle cell disease, thalassemia, HS and metabolic defects of RBCs. Finally, we describe the influence of shedding of nano-sized membrane vesicles from the RBC, the oxygenation state of hemoglobin and adaptive responses (such as exercise and high-altitude) on RBC deformability. Increased shedding of RBC vesicles, for example, is a feature of various RBC pathologies and vesicles are increasingly being considered to be a novel biomarker of RBC disorders (Pattanapanyasat et al., 2004; Nantakomol et al., 2012; Alaarg et al., 2013). They are considered to be involved in thrombosis and hemostasis (Biro et al., 2003; Livaja Koshiar et al., 2014) and associated with reduced RBC deformability (Waugh et al., 1992; Bosch et al., 1994).

## RBC Deformability In Hereditary Hemolytic Anemia

Anemia is considered to be hemolytic when RBCs are prematurely cleared from the circulation. Hemolytic anemia can be further subdivided into intra- or extravascular hemolytic anemia, and the underlying cause can be either inherited or acquired. Intravascular hemolysis is, as the name suggests, lysis of RBC in the vasculature. The cause can be hereditary, as seen in sickle cell disease (Pauling and Itano, 1949; Kato et al., 2017), but intravascular hemolysis can also be initiated by certain drugs (Cappellini and Fiorelli, 2008), by mechanical stress (for example through shear forces generated by artificial heart valves), by cold-agglutination (Körmöczi et al., 2006) or as a result of exhaustive exercise (Jordan et al., 1998). Intravascular hemolysis causes the release of hemoglobin into the plasma. Free hemoglobin is toxic and can lead to various clinical manifestations, such as hemoglobinuria, renal dysfunction, pulmonary hypertension and platelet activation (Rother et al., 2005).

Extravascular hemolysis is directly related to reduced RBC deformability. RBCs with reduced deformability fail to pass the spleen, which acts as an RBC quality-control organ (Mebius and Kraal, 2005; Deplaine et al., 2010). The red pulp of the spleen contains narrow inter-endothelial slits (MacDonald et al., 1987). Failure to pass through these narrow slits (Mebius and Kraal, 2005) leads to the uptake and breakdown of RBCs by macrophages (Burger et al., 2012). A number of hereditary RBC disorders result in reduced RBC deformability, which, as a consequence, leads to premature removal of RBCs in the spleen. Removal of RBCs by the spleen is, however, not only dependent on reduced deformability, but also occurs after recognition by macrophages. Senescent RBCs can be recognized and phagocytized by macrophages in the spleen upon binding of autologous antibodies to band 3 (Kay et al., 1983; Kay, 1984), exposure of conformational altered CD47 (Burger et al., 2012) or exposure of PS (Boas et al., 1998).

Hereditary forms of hemolytic anemia can affect the RBC membrane (i.e., HS, elliptocytosis, and pyropoikilocytosis) (Gallagher, 2004a; Perrotta et al., 2008; Da Costa et al., 2013), its metabolism (i.e., enzymopathies) (Zanella and Bianchi, 2000; van Wijk and van Solinge, 2005; Koralkova et al., 2014), cell hemoglobin (i.e., sickle cell anemia, unstable hemoglobin variants) (Higgs et al., 2012; Ware et al., 2017), or cellular hydration (i.e., HS, hereditary xerocytosis or Gardos Channelopathy) (Vives Corrons and Besson, 2001; Albuisson et al., 2013; Andolfo et al., 2013, 2015; Beneteau et al., 2014; Faucherre et al., 2014; Glogowska et al., 2015; Fermo et al., 2017a). While the primary genetic causes of these disorders are often well determined, less is known about the factors triggering the actual hemolysis. Striking examples are sickle cell disease, thalassemia, HS and the metabolic disorders of the RBC. All have well-known and well-studied primary genetic and molecular defects. However, little is known about the secondary mechanisms that may decrease RBC deformability and thereby contribute to the premature removal of these affected RBCs.

For example, the homozygous single point mutation in the *HBB* gene, substituting glutamic acid for valine at position 6 leads to sickle cell disease (Ware et al., 2017). Although the discovery that these RBCs tend to sickle at low oxygen tension was already provided by Hahn and Gillespie (1927), Kenney et al. (1961), it took decades to unravel the processes that contribute to sickle cell dehydration (Lew and Bookchin, 2005; Ataga et al., 2008), decreased deformability and increased endothelial cell adhesion (Alapan et al., 2015, 2016). We still do not understand how does a point mutation in hemoglobin beta chain cause these secondary pathological alterations in density and adhesiveness and how all these changes in turn impact the disease symptoms of the patient. Other examples are the enzymopathies, which are caused by mutations in, for example, PK or HK and lead to a shortage of metabolic energy inside the RBC. Again, although the primary effect on decreased levels of ATP in these diseases is well understood (Zanella et al., 2007), secondary processes such as altered K^+^ fluxes (Nathan et al., 1965) and phosphorylation of intracellular proteins (Thali et al., 2010; Wang et al., 2010), need further elucidation.

Moreover, for a substantial number of patients with hereditary hemolytic anemia – the primary causes of disease remain unknown, or were only identified very recently. For these patients, a comprehensive understanding of both primary and secondary defects of the affected RBCs is still lacking. This is illustrated by the recently discovered mutations in patients with hereditary xerocytosis, also known as dehydrated stomatocytosis (Andolfo et al., 2013). In these patients, stomatocytes are typically found in peripheral blood smears. However, the primary molecular cause for the anemia and morphological change of the RBC remained unknown for a long time, let alone that the secondary processes that are involved in the disease process were elucidated. Only recently, it was shown that hereditary xerocytosis was caused by mutations in genes for the mechanosensitive *PIEZO1* channel (Andolfo et al., 2013; Shmukler et al., 2014; Glogowska et al., 2017). Very recently, a mutation in the calcium-activated potassium channel subfamily N member KCNN4 (encoded by the human KCa3.1 gene, and also known as the Gardos channel in RBCs) (Andolfo et al., 2015; Glogowska et al., 2015; Rapetti-Mauss et al., 2015; Fermo et al., 2017a) was shown to cause ‘Gardos channelopathy’ a disease resembling yet at some points different from hereditary xerocytosis (Fermo et al., 2017a). Only then, the molecular diagnosis of these diseases was established in these patient groups and new insight on RBC deformability and RBC ion homeostasis with respect to ion channel function were obtained. The secondary processes that contribute to RBC clearance in these diseases are now being explored (Fermo et al., 2017a).

## Techniques to Determine RBC Deformability

**FIGURE 1:**
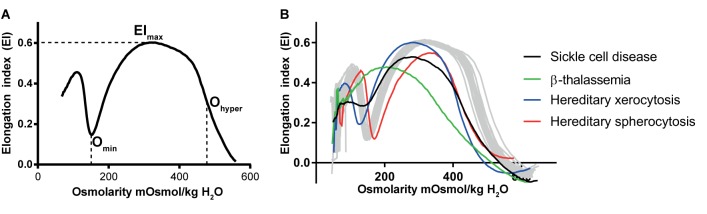
Osmotic gradient ektacytometry as a tool to measure red blood cell (RBC) deformability. The technique is discussed in detail by Clark et al. (1983); Lazarova et al. (2017), and Da Costa et al. (2016). Briefly, during osmotic gradient ektacytometry, the RBC is subjected to an osmotic gradient (range ≈50 mOsmol/kg H_2_O – 650 mOsmol/kg H_2_O) under constant shear stress, while the elongation index (EI) is measured. The EI corresponds to the deformability at various osmotic conditions. **(A)** the left graph depicts an osmotic gradient ektacytometry curve from a healthy control with various intersection points: the EI_max_ reflects the maximal deformability of the RBC, *O*_min_ reflects the osmotic fragility and *O*_hyper_ reflects the (de)hydration state (or intracellular viscosity) of the RBC. **(B)** In the right graph osmotic gradient curves of patients with sickle cell disease (black line), β-thalassemia (green line), hereditary xerocytosis (blue line) and hereditary spherocytosis (red line) and several individual healthy controls (gray lines, *n* = 20) are depicted.

A currently well-established tool to probe RBC deformability is osmotic gradient ektacytometry, which is routinely used in the diagnosis of patients with hereditary hemolytic anemia. The technique is performed on a Laser-assisted Optical Rotational Cell Analyzer (Lorrca). It assesses RBC deformability, osmotic fragility and cellular hydration status (see **Figure 1**) (Clark et al., 1983; Da Costa et al., 2016; Lazarova et al., 2017; Llaudet-Planas et al., 2017). In osmotic gradient ektacytometry, the maximum RBC deformability is represented by the maximum elongation index (EI_max_). The *O*_min_ represents the osmotic value (mOsmol/kg H_2_O) where the elongation index (EI) is minimal, corresponding to the 50% lysis point as determined by the classical osmotical fragility test (Clark et al., 1983). The hydration status (or intracellular viscosity) is represented by *O*_hyper_ (mOsmol/kg H_2_O). The value corresponds with the hypertonic osmolarity where the EI is 50% of EI_max_. The value of these three parameters as well as the shape of the curve is used in the diagnosis of various disorders of the RBC membrane and hydration station (**Figure 1B**).

*O*_hyper_ correlates with the reciprocal function of the MCHC (Clark et al., 1983). In healthy individuals, during RBC aging increased MCHC is observed and this correlates with decreased RBC deformability (Clark et al., 1983; Bosch et al., 1994). A similar observation has been made for RBCs from patients with sickle cell disease and HS. Also here, a decrease in RBC hydration (i.e., decreased *O*_hyper_ and increased MCHC) of patient’s cells is associated with a decrease in the maximum RBC deformability (EI_max_) (Clark et al., 1983; Bunn, 1997).

Besides osmotic gradient ektacytometry, there are other methods to measure RBC deformability (Tomaiuolo, 2014). A number of techniques that are also used in diagnostics measures RBC deformability as a function of shear stress, for example the RheoScan-D (Shin et al., 2007), Automated Rheoscope and Cell Analyser (ARCA) (Dobbe et al., 2002) and the deformability measurement module on the Lorrca (Hardeman et al., 1987). The results of RheoScan-D and the deformability measurements (shear-stress module) on the Lorrca were observed to be comparable (Shin et al., 2007). However, RheoScan-D measures RBC deformability under microfluidic conditions after the application of shear stress, while shear-stress on RBCs in the deformability measurements on the Lorrca requires a larger set-up with a rotating cup (Hardeman et al., 1987; Shin et al., 2007). The ARCA measures RBC deformability with a microscope after the application of shear-stress, and therefore has the potential to measure the RBC deformability of subpopulations (Dobbe et al., 2002).

Most direct measurements of single cells include: micropipette (Waugh et al., 1992), atomic force spectroscopy and holographic optical tweezers (HOT) (Steffen et al., 2011; Kaestner et al., 2012). The mechanical probes and the optical approach are complementary since 30 pN is a lower force limit for the mechanical measurements and an upper limit for the HOT in the context of investigating RBCs (Minetti et al., 2013). Currently microfluidic approaches becoming increasingly popular to investigate RBC deformability (Cluitmans et al., 2014; Guo et al., 2014; Picot et al., 2015; Danielczok et al., 2017). While the latter method has the potential to become a routine diagnostic tool, the former single cell methods are mostly dedicated to basic research due to their instrumental complexity.

A further very simple method to measure RBC deformability is the readout of RBC filterability, e.g., by measuring RBC passage through cellulose columns (Oonishi et al., 1997). Unfortunately there is currently no standardized column/readout system on the market that would allow comparison of filterability between diagnostic laboratories. In addition, RBC passage through cellulose columns or filters is highly subjected to changes in cellular volume (such as MCV), which may affect results. A summary of available techniques to measure RBC deformability is depicted in **Table 1**.

**Table 1:** Overview of techniques to measure RBC deformability.

Method	Principle	Readouts	Advantages	Disadvantages/sources of mistake
Osmotic fragility test (OFT) (Parpart et al., 1947)	The OFT tests RBC lysis at various osmotic conditions. Lysis in OFT depends on critical RBC volume needed for hemoglobin content.	50% lysis point	Relatively easy technique	Requires pre-incubation at 37°C and heparin blood, measurement of population average. pH and temperature of solutions can affect results. Low sensitivity (Bianchi et al., 2012).
Osmotic gradient ektacytometry (Clark et al., 1983; Da Costa et al., 2016; Lazarova et al., 2017)	RBC deformability is constantly measured under constant shear stress while osmotic gradient gradually increases from hypotonic to hypertonic conditions.	Maximum deformability (EI_max_), RBC hydration (*O*_hyper_) and 50% lysis point in OFT (*O*_min_)	Robust and reproducible, diagnostic technique, enables comparison between laboratories	Measures RBC population average
Shear-stress dependent deformability (Hardeman et al., 1987; Dobbe et al., 2002; Shin et al., 2007)	Measurement of RBC deformability after application of constant or increasing levels stress-stress (i.e., Automated Rheoscope and Cell Analyser (ARCA), RheoScan-D, or deformability on Lorrca).	Elongation index (EI) and maximum deformability EI_max_	Easy, reproducible and diagnostic techniques Automated Rheoscope and Cell Analyser (ARCA) can measure deformability of subpopulations	RheoScan-D and deformability measurements on Lorrca measures populations average. Automated Rheoscope and Cell Analyser (ARCA) requires a microscope, which leads to number of cells that are out of focus and are rejected from analysis
Atomic force microscopy (AFM) (Yeow et al., 2017)	Scanning technique that uses a tip as a probe to detect morphological changes and changes in deformability. Measures the resistance after application of force.	Elasticity, force, resistance	Single cell technique and AFM can measure RBC deformability at different spots per RBC. Topographic images can be obtained.	Advanced equipment needed and labor extensive, no gold standard
Micropipette (Waugh et al., 1992)	Aspiration of cells or membrane parts.	Surface area, volume, sphericity, elasticity	Single cell technique	Labor intensive
Holographic optical tweezers (HOT) (Steffen et al., 2011; Kaestner et al., 2012)	Usage of highly focused to generate laserbeams to with attractive or repulsive forces.	Depending on set-up; fluidity, elongation, fluorescence microscopy read-out [e.g., Ca^2+^ (fluo-4-AM)]	Low forces can be applied	Advanced equipment needed and labor extensive, no gold standard
Microfluidics (Cluitmans et al., 2014; Guo et al., 2014; Danielczok et al., 2017; Picot et al., 2015)	RBC suspension is injected in network of microfluidic channels. In these channels RBC are subjected to small pores where RBC deformability is essential.	Depends on microfluidic set-up	Measurement of individual RBCs, RBC deformability measured under relative physiological conditions, various set-ups available	Technique still in development phase, no gold standard available, which hampers comparison between laboratories and reduces interlaboratory reproducibility. RBCs can block microfluidic device
RBC filterability (Oonishi et al., 1997)	RBC needs to pass filter with various pore-sizes. Also various columns are used with different particle and particle sizes (e.g., cellulose, metal beads).	Dependent on set-up: time, lysis, resistance	Relatively easy technique	No standardization available and no standardized commercial technique available. No comparison between laboratories possible. Can be affected by parameters such as MCV.


## Determinants of RBC Deformability

### RBC Hydration

#### Regulation of RBC Hydration

**FIGURE 2:**
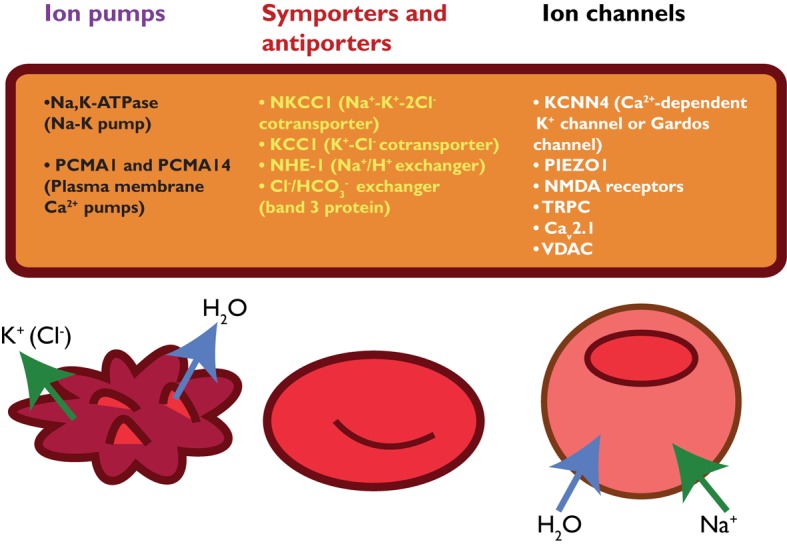
schematic overview of RBC ion pumps (Sachs, 2003; Tiffert et al., 2003), symporters and antiporters (Bernhardt et al., 1988; Wolfersberger, 1994) and ion channels (Bouyer et al., 2012; Kaestner, 2011, 2015).

Red blood cell deformability is highly influenced by RBC volume control and by ion content, both regulated by ion pumps, ion channels, symporters and antiporters (Gallagher, 2013) (see **Figure 2**). When ion channels are open, ions move following their electrochemical gradients, while ion pumps can actively move these ions against the gradient (Gadsby, 2009). Symporters and antiporters may also create secondary ion gradients, but require the pre-existing gradients for at least one type of ions as a driving force to transport the other ion types against the gradient. Symporters transport two (e.g., K^+^-Cl^-^ cotransporter) or more (e.g., Na^+^-K^+^-2Cl^-^ cotransporter) ions in the same direction using driving force for one of them, while antiporters (e.g., Na^+^/H^+^ exchanger or anion exchanger) exchange two ions that move in the opposite direction (Wolfersberger, 1994). These transporters, pumps and channels are crucial in resisting/adapting to local osmotic changes and maintaining RBC volume (Wieth, 1979; Liu et al., 2011; Thomas et al., 2011; Gallagher, 2013; Lew and Tiffert, 2017). RBC volume changes result from differences in osmotic pressure (Sugie et al., 2018), which results in water transport by aquaporins of which AQP1 and AQP3 are found in human RBCs (Yang et al., 2001). Cotransport of water is, however, also possible through the K^+^-Cl^-^ cotransporter (KCC) (Zeuthen and Macaulay, 2012). Volume changes are also associated by in- and efflux of several amino acids and amino-acid derivates, such as taurine (Goldstein and Brill, 1991; Goldstein and Davis, 1994).

As mentioned previously, MCHC is a parameter that reflects the RBC hydration state, and is dependent on the hemoglobin concentration in RBCs, RBC volume, RBC membrane loss and water content. Quick movements of ions and water result in acute changes in MCHC whereas long-term effects on MCHC are often associated with release of hemoglobin-free vesicles. RBC hydration may be quantified using the *O*_hyper_ value as obtained by osmotic gradient ektacytometry (see **Figure 1** and see section “Techniques to Determine RBC Deformability”). Changes in RBC density detected using cell distribution within Percoll density gradients reflects both membrane loss and the changes in RBC surface-to-volume ratio and alterations in intracellular ion and water content. In-depth morphological analysis could be used as a complementary approach to discriminate between membrane and ion/water loss.

#### Primary Changes of RBC Hydration

In this section we shall address hereditary hemolytic anemias associated with the changes in the intracellular ion and secondary to the latter changes in water content. One of the players in this type of disorder is a mechano-sensitive PIEZO1 channel, a non-selective cation channel permeable for Ca^2+^ that in turn can activate the calcium activated K^+^ channel subfamily N member KCNN4 (or Gardos channel in RBCs) (Kaestner et al., 2018). Thus, hydration state is largely dependent on the shear stress intensity and the hydration state of RBCs from splenectomized and non-splenectomized patients with the same mutation may vary substantially. Furthermore, hydration state is a function of RBC age, young RBCs being more hydrated than the senescent cells (Clark et al., 1983; Lutz et al., 1992).

A RBC disorder characterized by primary hydration changes is hereditary xerocytosis (HX), also known as dehydrated stomatocytosis. HX is a form of hereditary hemolytic anemia, and patients often suffer from iron-overload (Zarychanski et al., 2012). In addition, in many RBC disorders splenectomy relieves symptoms and increases RBC survival, but in HX, splenectomy is contra-indicated as it drastically increases the risks for thrombotic events (Stewart et al., 1996; Jaïs et al., 2003; Iolascon et al., 2017). HX was related to mutations in the mechanosensitive PIEZO1 channel (Zarychanski et al., 2012; Albuisson et al., 2013; Li et al., 2014). PIEZO1 mutations lead to stabilization of the “active” state or destabilization of the “inactive” state of the PIEZO1 protein (Albuisson et al., 2013). These alterations in channel function lead to increased intracellular levels of Ca^2+^ and subsequent KCNN4 (Gardos channel) activation (Cahalan et al., 2015; Danielczok et al., 2017). KCNN4 activation leads to efflux of K^+^ and dehydration. The relatively low intracellular levels of K^+^ could therefore serve as a biomarker for HX (Zarychanski et al., 2012; Gallagher, 2017). Although PIEZO1 mutations leads to KCNN4 (or Gardos channel) activation, RBCs with mutations in KCNN4 (Gardos channelopathy) exhibit a different pathology (Fermo et al., 2017a). For example, HX caused by mutations in PIEZO1 lead to severe RBC dehydration, which is also shown by a left-shift of *O*_hyper_ in the osmotic gradient curve (see **Figure 1B** and see section “Techniques to Determine RBC Deformability”) while mutations in KCNN4 (or Gardos channel) may cause only slight alterations in the osmotic gradient curve (Fermo et al., 2017b). In addition, RBCs from patients with KCNN4 mutations were found to exhibit increased activity of Na-K-ATPase that is most likely triggered by the increase in the intracellular Na^+^ concentrations. The hyperactivated Na, K-pump compensates for the dissipation in Na/K gradients and pumps K^+^ back into the cells, but fails to restore normal K^+^ content completely (Fermo et al., 2017a). Currently, however, it is difficult to measure intracellular water content or RBC volume directly and accurately enough, which makes it challenging to discriminate between water loss and membrane loss.

### Cytoskeletal Network

#### Primary Changes of the Cytoskeletal Network

In addition to the protein defects, described above, that directly disturb the membrane and thereby the ability of the RBC to deform there are also secondary causes affecting membrane (protein) function and, thereby, RBC deformability. Examples of these are membrane protein phosphorylation, RBC density and RBC vesiculation.

The RBC membrane is built from a basic triangular network of α- and β-spectrin molecules connected to band 3, ankyrin and protein 4.1 (Perrotta et al., 2008; Salomao et al., 2008). This network provides the RBC with a certain stability and simultaneously the ability to deform. HS (HS) is characterized by defects in proteins forming the cytoskeletal network or proteins forming ankyrin and junctional complexes. This usually concerns mutations in band 3, protein 4.2, α-spectrin, or ankyrin (Perrotta et al., 2008). These mutations lead to membrane instability, membrane loss through release of hemoglobin-free vesicles, with consequent decreased surface area-to-volume ratio, increase in MCHC and density and formation of spherocytes (Perrotta et al., 2008). Spherocytes show a characteristic loss of RBC deformability, leading to premature removal of these RBCs from the circulation (Perrotta et al., 2008). Hereditary elliptocytosis (HE) is a disorder characterized by disturbances of the horizontal cytoskeletal interactions and are usually caused by mutations in α-spectrin, β-spectrin and protein 4.1R. As the name suggests, the disturbed horizontal interactions lead to the formation of elliptocytes. The majority of HE patients are clinically asymptomatic, but few patients suffer from hemolysis, jaundice and splenomegaly (Da Costa et al., 2013).

#### Secondary Disturbances of the Cytoskeletal Network

The triangular spectrin network, connected through ankyrin and protein 4.1R to band 3 supports membrane stability and contributes to RBC flexibility and deformability. Tyrosine phosphorylation of in the individual proteins regulates interaction forces between the elements of this network. Tyrosine phosphorylation is regulated by phosphotyrosine kinases and by phosphotyrosine phosphatases. Under normal conditions, protein regulatory tyrosine residues are only phosphorylated to a limited degree (Terra et al., 1998; Zipser et al., 2002). In the RBC membrane, phosphorylation of protein 4.1R, for example, leads to a reduced membrane stability (Muravyov and Tikhomirova, 2013), and phosphorylation of ankyrin regulates its binding to band 3 (Muravyov and Tikhomirova, 2013). The dissociation constant (*K_d_*) between ankyrin and band 3 influences RBC deformability without a loss in elasticity, underlining that cytoskeletal protein phosphorylation has implications on RBC deformability (Anong, 2006).

The effect of phosphorylation of band 3, the most abundant membrane protein, on RBC deformability is not entirely clear. The possible physiological relevance of band 3 protein Tyr phosphorylation state emerges from the fact that it is controlled by hormones such as insulin (Marques et al., 2000) and that it is altered in patients with hemoglobinopathies (Terra et al., 1998). Saldanha et al. (2007) showed that dephosphorylation of band 3 Tyr residues does not affect RBC deformability. It, therefore, appears that phosphorylation of band 3 does not affect RBC deformability directly, but may alter band 3 protein aggregation and its interaction with extracellular matrix components.

Lopes de Almeida et al. (2012) showed that high concentrations of fibrinogen in combination with dephosphorylated band 3, leads to mildly increased RBC deformability at low shear stress. In contrast to these findings, Reid et al. (1984) report decreased RBC deformability in diabetic patients with hyperfibrinogenemia in absence of dephosphorylating agents (Reid et al., 1984). The decreased RBC deformability in the absence of dephosphorylating agents seems to underline that phosphorylation is not involved. However, insulin is implicated in the activation of tyrosine kinases that in turn phosphorylate band 3. In diabetes types I and II, glucose and insulin homeostasis is disturbed leading to increased protein tyrosine phosphatase activities and aberrant band 3 phosphorylation patterns (Marques et al., 2000), potentially leading to reduced RBC deformability and increased RBC turnover. This same principle may also play a role in patients with sepsis, where investigators have shown that tyrosine phosphorylation of band 3 was increased in septic mice and that this increase was accompanied by reduced RBC deformability (Condon et al., 2007).

Band 3 phosphorylation was found to be increased by a rise in intracellular Ca^2+^-concentrations due to dissociation of phosphotyrosine phosphatase from band 3 (Zipser et al., 2002). Interestingly, sickle cell disease is characterized by an increase in both intracellular Ca^2+^ concentration (Bogdanova et al., 2013) and tyrosine phosphorylation of band 3 (Terra et al., 1998), which potentially could thus influence RBC membrane stability. The importance of band 3 phosphorylation for RBC shape and deformability is highlighted by a number of *in vitro* experiments. For example, morphological changes of RBCs were observed after Tyr-phosphorylation of band 3 mediated by pervanadate (Bordin et al., 1995). In contrast, Ser/Thr-phosphorylation of spectrin, which was selectively induced by okadaic acid did not result in morphological changes (Bordin et al., 1995). Unfortunately, the deformability of RBCs after pervanadate and okadaic treatment was not measured in these studies, but it seems reasonable to assume that RBC deformability is affected since both membrane protein phosphorylation and RBC morphology have been associated with altered RBC deformability (Reid et al., 1984; Chabanel et al., 1987; Hasegawa et al., 1993; Saldanha et al., 2007; Lopes de Almeida et al., 2012).

#### RBC Hydration Changes Associated With Cytoskeletal Defects

Although reduced RBC deformability in HS mainly results from cytoskeletal disturbances (Perrotta et al., 2008), spherocytes experience dehydration and increased leakage of K^+^ (De Franceschi et al., 1997; Gallagher, 2017). To compensate for this increased K^+^ leakage, spherocytes are also found to have increased activity of the Na-K pump and the NKCC1 (also known as the Na-K-2Cl cotransporter, coded by gene SLC12A2) (Vives Corrons and Besson, 2001). RBCs of transgenic mice deficient for ankyrin or spectrin show increased activity of the Na-K pump, but normal activities of the NKCC1 and K^+^-Cl^-^ cotransporters (Peters et al., 1999). This contrasts with protein 4.2-deficient mice, where Na-K pump activity is normal, while activities of NKCC1 and K^+^-Cl^-^ cotransporters are increased (Peters et al., 1999). These differences in pump activities between protein 4.2 deficient and spectrin- or ankyrin-deficient mice support the concept that membrane proteins interact with ion channels of the RBC.

The RBC dehydration in HS also involves increased intracellular Ca^2+^ levels (Hertz et al., 2017). Increased intracellular Ca^2+^ correlates with decreased RBC deformability and RBC membrane stiffening (Bogdanova et al., 2013). For example, after *in vitro* ATP-depletion of HS RBCs, Ca^2+^ influx is increased compared to healthy RBCs (Shimoda et al., 1984). The detailed mechanisms beyond this Ca^2+^ influx have up to now not been identified and may include PIEZO1 or NMDA receptors both of which are activated by shear stress in “stiff” less deformable cells.

In protein 4.2^-/-^ mice, displaying the HS phenotype, KCNN4 (or known as Gardos channels in RBCs) were noted to be functionally up-regulated, or at least more KCNN4 activity was measured (Peters et al., 1999; De Franceschi et al., 2005). This can be considered as a protective mechanism that leads to an outward flux of K^+^ and water from RBCs. This mechanism may compensate for the decreased surface-to-area ratio. Indeed, increased lysis was observed when these RBCs were exposed to KCNN4 (or Gardos-channel) inhibitors (De Franceschi et al., 2005). These findings indicate that KCNN4 is activated in HS in order to facilitate RBC dehydration, which maintains hemoglobin in RBCs at the expense of water. Recent research confirms the finding that RBC dehydration and formation of denser RBCs may protect the RBC in HS from lysis and premature uptake. We observed that mild HS is accompanied with prolonged RBC life-span, relatively mild reductions in RBC deformability and denser RBCs when compared with RBCs from patients with moderate/severe HS (Huisjes et al., 2017a). Most likely, the mild reduction in RBC deformability in patients with mild HS avoids premature splenic uptake and thus may provide the HS RBC time to lose membrane and to become dense.

### Red Cell Metabolism

#### Primary Changes of Red Cell Metabolism

**FIGURE 3:**
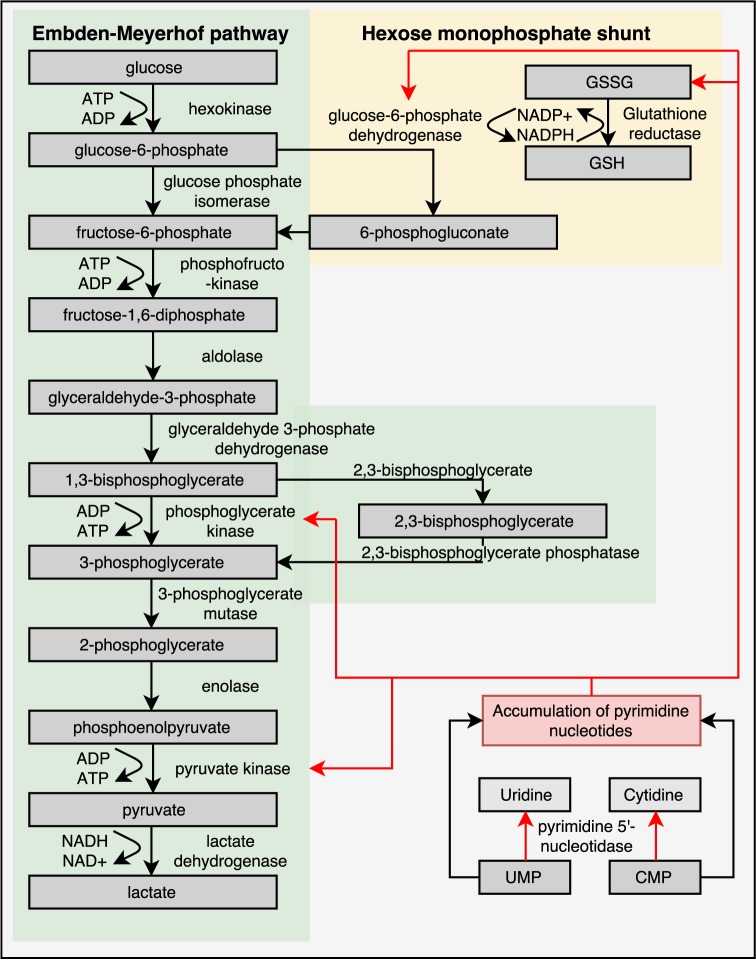
Glycolytic pathway of RBCs is essential for energy metabolism as RBCs lack mitochondria and a nucleus. Enzyme deficiencies can lead to decreased intracellular ATP levels, which possibly reduce RBC deformability. Depicted here are the Embden–Meyerhof pathway, hexose monophosphate shunt and the influence of a pyrimidine 5′-nucleotidase deficiency. Red arrows illustrate negative feedback or reduced production. This illustration is a merged figure adapted from both van Wijk and van Solinge (2005) and Zanella et al. (2006).

Mature RBCs of healthy individuals lack mitochondria and are therefore entirely dependent on the glycolytic pathway for the production of energy in the form of ATP. In the glycolytic pathway, glucose is converted to lactate by several enzymatic steps (**Figure 3**) (van Wijk and van Solinge, 2005; Koralkova et al., 2014). Key regulatory enzymes in the glycolytic pathway are HK, phosphofructokinase and PK. Hereditary metabolic disorders, or enzymopathies, are disorders impairing cellular energy production and balance, in particular ATP production. ATP is essential in the regulation of RBC deformability, viability, regulatory cascades involving phosphorylation and activity of ion transport involving Na, K-ATPase, and Ca ATPase (Weed et al., 1969; Tuvia et al., 1998; Martinov et al., 2000; Fischer et al., 2003; Park et al., 2010; Makhro et al., 2016).

Adenosine triphosphate plays a key role in maintenance of ion gradients and should impact cytoskeletal structure and RBC shape (Tuvia et al., 1998; Park et al., 2010). However, glycolytic enzymopathies usually do not show altered RBC morphology (Koralkova et al., 2014; Huisjes et al., 2017b). For reasons that are not yet well understood, metabolically defective RBCs are prematurely removed by the spleen, causing chronic Hereditary Non-Spherocytic Hemolytic Anemia (HNSHA) (Koralkova et al., 2014).

Several observations point to mechanisms that may be involved in the premature clearance. RBCs from a patient with pyruvate kinase deficiency (PKD) were found to leak K^+^ more rapidly and were found to consume ATP at an accelerated rate (Nathan et al., 1965). This most likely contributes to the decreased RBC viability in this disease and could result in reduced RBC deformability (Leblond et al., 1978). A possible explanation for increased clearance of RBCs with metabolic defects is the imbalance in Ca^2+^ uptake and extrusion (Bogdanova et al., 2013; Hertz et al., 2017). In agreement with the hypothesis that ATP deprivation will cause Ca^2+^ overload due to the inability to actively extrude Ca^2+^ from the cells by the Ca^2+^ pumps, decreased ATP levels in RBCs with metabolic defects were associated with abnormally high intracellular Ca^2+^ and exposure of PS, an important cellular ‘eat-me’ signal (de Back et al., 2014). Alternatively, defective metabolism may impact the redox state of RBCs making them more susceptible to oxidative stress (Cappellini and Fiorelli, 2008).

Despite the intuitive feeling that intracellular ATP levels should correlate with cellular deformability, evidence for this is so far inconclusive. This may be related to the techniques used. For example, research performed by Karger et al. (2012) did not show any correlation between intracellular ATP and deformability in RBC-concentrates intended for transfusion when assessing deformability by increasing shear stress. On the other hand, studies using viscosity and filterability assays did find correlations between intracellular ATP and deformability and showed that deformability is indeed dependent on intracellular ATP levels (Weed et al., 1969; Fischer et al., 2003). Atomic force microscopy studies also revealed increased membrane stiffness after ATP depletion due to reduced spectrin phosphorylation (Picas et al., 2013). We hypothesize that the different results from these experiments are possibly caused by the fact that ATP is not required for RBCs to elongate after the application of external forces, such as shear stress but ATP might be essential in the process of RBC deformation toward different shapes, as seen in filterability measurements.

#### Secondary Changes of Metabolism

**FIGURE 4:**
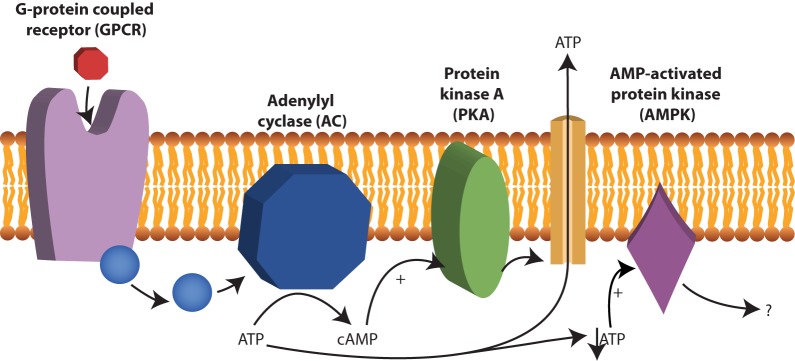
cAMP production by adenylyl cyclase (AC). AC is activated after stimulation by G-protein coupled receptors (GPCR) and converts ATP to cAMP. Examples of GPCRs 2-adrenergic receptor, the lysophosphatidic β in RBCs are the erythrocyte acid (LPA) receptor and the purinergic (P2Y) receptor. cAMP acts as a second messenger in RBCs and among other things stimulate protein kinase A (PKA). Under low ATP concentrations, AMP-activated protein kinase (AMPK) becomes active and can inactivate or activate yet not fully understood processes in RBCs.

Cyclic AMP (cAMP) is a second messenger and is derived from ATP. In nucleated human cells, cyclic AMP (cAMP) is synthesized from ATP by adenylate cyclase (AC) upon stimulation by G-protein coupled receptors (GPCR) and there is evidence that this process also occurs in human RBCs (Oonishi et al., 1997; Tuvia et al., 1999; Sprague et al., 2006; Kostova et al., 2015) (see **Figure 4**). Examples of GPCRs on RBCs are the erythrocyte β2-adrenergic receptor (Harrison et al., 2003), the lysophosphatidic acid (LPA) receptor (Wang et al., 2013) and the purinergic (P2Y) receptor (Kostova et al., 2015). The activation of the erythrocyte β2-adrenergic receptor by catecholamines such as epinephrine (or adrenaline) affects RBC deformability (Tuvia et al., 1999). *In vitro* experiments with increased concentration of adrenaline, lead to increased membrane fluctuations and increased RBC filterability (Tuvia et al., 1999).

##### Intracellular mechanisms that sense and control cellular energy status in RBCs

**FIGURE 5:**
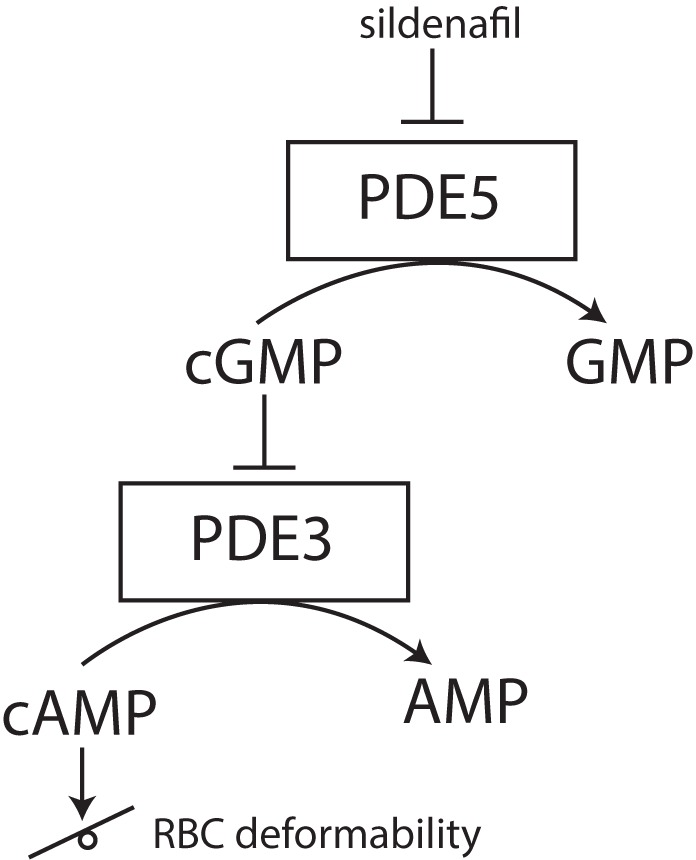
effect of PDE-5 inhibitors, such as sildenafil, on RBC deformability. PDE-5 inhibitors elevate intracellular concentrations of cGMP, thereby indirectly inhibiting PDE3. Inhibition of PDE3 does raise the intracellular concentration of cAMP. Depending on the degree of this intracellular cAMP elevation, RBC deformability can be either increased or decreased.

Phosphodiesterases tightly regulate levels of cAMP in RBCs (Adderley et al., 2011). PDEs catabolize the phosphodiesters in cyclic nucleotides, such as cAMP to AMP or cGMP to GMP. By inhibiting these PDEs, cyclic nucleotides remain intact and active. The reports of cAMP effects on RBC deformability are, however, ambivalent. Recently it was found that sildenafil, which is a PDE inhibitor and well-known for its function in the treatment of erectile dysfunction and pulmonary hypertension, has beneficial effects on RBC deformability in sickle cell disease at low concentrations *in vitro*. Increase in nitric monoxide bioavailability was suggested to improve RBC deformability (Grau et al., 2013; Kuhn et al., 2017). However, increased concentrations of sildenafil impaired sickle RBC filterability (Hagley et al., 2011). Typically, inhibition of cGMP-specific PDE type 5 (PDE-5) by sildenafil reduces the degradation of the second messenger cGMP to GMP and increases smooth muscles relaxation. In RBCs, this PDE-5 inhibition increases not only intracellular levels of cGMP but also preserves intracellular levels of cAMP via the inhibitor activity of cGMP on PDE-3 (see **Figure 5**) (Adderley et al., 2010; Knebel et al., 2013). The effect of increased cAMP levels was also studied using a spleen-like microfiltration system. Increased retention in the microfiltration system, reflecting decreased deformability, was observed after incubation of RBCs with sildenafil (Ramdani et al., 2015). Moreover, the production of cGMP from GTP is synthesized by guanylate cyclase (Derbyshire and Marletta, 2012). Guanylate cyclase is activated by nitric oxide (NO) and the addition of sodium nitroprusside to RBCs as an artificial nitric oxide donor does prevent Ca^2+^ influx and Ca^2+^-mediated stiffness of RBCs (Barodka et al., 2014).

In addition, the effect of cAMP on the release of ATP is still under debate. A role for cAMP in the release of ATP to its extracellular environment via PKA was proposed (Sprague et al., 2007; Adderley et al., 2011; Knebel et al., 2013) (see **Figure 4**). Others, however, have attributed the release of ATP solely to hemolysis of the RBC (Sikora et al., 2014) or to release via transporters such as Pannexin 1 (Locovei et al., 2006) or PIEZO1 (Cinar et al., 2015).

Recently AMPK was also found to be present in RBCs (Thali et al., 2010). The exact function of AMPK in RBCs is unknown, however, in other cells, AMPK is essential to balance ATP consumption and ATP production. In muscles and fat AMPK regulates glucose uptake (Mihaylova and Shaw, 2011). Likely, AMPK also regulates these processes in RBCs, as RBCs from AMPK^-/-^ mice were less viable after *in vitro* glucose depletion (Föller et al., 2009). Interestingly, these AMPK^-/-^ mice had increased reticulocytes count, decreased RBC deformability, increased osmotic fragility, increased spleen size and decreased RBC lifespan (Foretz et al., 2010; Wang et al., 2010). This indicates a significant contribution of AMPK in maintaining RBC function and viability. AMPK is an energy-sensing enzyme, which is activated by low AMP: ATP ratios and inhibited by high AMP: ATP ratios (Wang et al., 2003). Under low ATP concentrations, AMP and ADP can bind to AMPK and change its conformation, thereby preventing the protein from activation by phosphorylation (Mihaylova and Shaw, 2011). Chemical agents that increase intracellular levels of cAMP are forskolin and isobutyl-methyl-xanthine (IBMX). Forskolin activates AC and thus leads to increased formation cAMP from ATP, while IBMX inhibits PDEs and maintains cAMP levels. Both forskolin and IBMX lead to AMPK inhibition, which reveals a molecular interaction between cAMP and AMPK activity (Hurley et al., 2006). This interaction could play a role in altered deformability in patients suffering from glycolytic enzymopathies. The low RBC ATP concentrations in these patients would lead to low levels of cAMP and increased intracellular levels of non-phosphorylated (inactive) AMPK.

Phosphorylation of AMPK may also be involved in G6PD-deficiency. G6PD deficiency is the most common RBC enzyme deficiency with over 400 million affected people (Cappellini and Fiorelli, 2008). Deficiency of this enzyme is associated with decreased ability of the RBC to withstand oxidative stress (Cappellini and Fiorelli, 2008; Mangat et al., 2014; Tang et al., 2015). Exposure of normal and G6PD-deficient RBCs to the oxidant diamide leads to a decrease in RBC deformability of the G6PD-deficient RBCs due to the inability of the RBC to maintain the intracellular glutathione-pool (Tang et al., 2015). Depletion of glutathione also leads to activation of AMPK by phosphorylation in G6PD-deficient RBCs. The activation of AMPK in G6PD-deficient RBCs after exposure to oxidative stress is most likely a consequence of ATP-consuming compensation mechanisms after excessive glutathione loss (Tang et al., 2015).

Protein kinase C alpha (PKCα) possibly also regulates RBC deformability. PKCα is involved in the phosphorylation of proteins in the RBC, such as adducin, protein 4.1R and protein 4.9 (George et al., 2010). Moreover, PKCα is involved in stimulation of glucose uptake by phosphorylation of the glucose transporter and participates in the activity of calcium channels (Kanno et al., 1995; George et al., 2010; Wagner-Britz et al., 2013). Consequently, phosphorylation and dephosphorylation of these proteins can be altered upon depletion of glucose. Glucose depletion was found to activate PKCα and this is accompanied by an increase in intracellular Ca^2+^ and, consequently, exposure of the membrane phospholipid PS (Klarl et al., 2006). The phosphorylation of adducin and protein 4.1R by PKCα could change RBC behavior and could influence RBC deformability (George et al., 2010) in a way similar to what is seen, for example, after phosphorylation of band 3 (Saldanha et al., 2007). Interestingly, homology analysis between murine, rat and human showed a conserved region in both liver- and RBC-specific PK for a PKCα binding site (Kanno et al., 1995). PKCα regulates RBC membrane stability and this conserved region for PK indicates a possible role for PK in this process (Kanno et al., 1995). In addition, PK converts phosphoenolpyruvate (PEP) to pyruvate (**Figure 3**) and previously it has been recognized that phosphorylation of PK by cAMP-dependent protein kinases leads to a reduced affinity of PEP for PK, which leads to a reduced production of ATP (Marie et al., 1980). The decreased cAMP and ATP production in metabolic disorders of the RBC, such as PK-deficiency, could subsequently result in disturbed RBC deformability (Leblond et al., 1978).

##### Role of purines and pyrimidines in RBC deformability

It has been suggested that pools of ATP are intracellular enclosed by the membrane proteins ankyrin, β-spectrin, band 3, and GAPDH, serving as substrates for both the Na^+^, K^+^-, and Ca^2+^ -pumps (Chu et al., 2012). This indicates an interplay between membrane proteins, energy stores and ion channel function of the RBC. Interestingly, the chemotherapeutic agent 5-FU was shown to induce changes in RBC rigidity, morphology and ion balance, most likely by altering ATP levels in the RBC (Spasojević et al., 2005). 5-FU is a pyrimidine antagonist (Longley et al., 2003) and since purine and pyrimidines play key roles in cellular metabolism and energy homeostasis. 5-FU could, therefore, influence RBC deformability. The activating and feedback mechanisms of purines and pyrimidines on glycolytic enzymes have previously been described by Seider and Kim (1979) and Tomoda et al. (1982). For example, in bovine RBCs incubated with glucose, adenosine stimulated ATP production compared to incubation with glucose alone. Most likely, adenine (i.e., the nucleobase of adenosine) stimulates HK activity (Seider and Kim, 1979). The effect of pyrimidines in RBCs has also been studied in two patients with a pyrimidine 5′-nucleotidase (P5N) deficiency, which lack this enzyme involved in clearance of pyrimidines from the RBC. P5N deficiency increases intracellular concentrations of pyrimidine nucleotides, which eventually leads to hemolytic anemia (Vives L Corrons, 2000). P5N-deficient patients were also found to have increased levels of reduced glutathione (Tomoda et al., 1982). At the same time, pyrimidine 5′-nucleotides (such as cytidine mono-, di-, and triphosphate (CMP, CDP, CTP) or uridine mono-, di-, and triphosphate (UMP, UDP, UTP)) decreased the activity of glucose-6-phosphate dehydrogenase (G6PD, **Figure 3**). As mentioned earlier G6PD activity is crucial in the anti-oxidative defense of RBCs. Indeed, RBCs from P5N-deficient patients were found to be more susceptible to oxidative stress, as reflected by increased formation of Heinz bodies (Tomoda et al., 1982), even despite increased reduced glutathione concentrations. Besides their role as a morphological marker of oxidative stress, Heinz bodies can lead to a decreased deformability by themselves (Hasegawa et al., 1993).

### Hemoglobin

#### Primary Changes of Hemoglobin

Hemoglobin is the main component of the RBC and responsible for the delivery and removal of oxygen and carbon dioxide to and from the tissues, respectively. Intracellular hemoglobin concentrations and its state (polymerization, crystallization, degradation, and oxidation) also defines cytosolic viscosity making up 19.9–22.3 mmol/L in cells of healthy humans. Hemoglobin is composed of two α- and β-hemoglobin molecules together composing a heterotetramer. Disorders of hemoglobin can be subdivided into hemoglobinopathies (e.g., sickle cell anemia) and thalassemias (α- and β-thalassemia) (Galanello and Origa, 2010; Harteveld and Higgs, 2010; Higgs et al., 2012; Ware et al., 2017).

Sickle cell anemia is caused by a single point mutation in the *HBB* gene at position 6 substituting glutamic acid to valine (HbS). This substitution causes formation of HbS polymers of deoxygenated hemoglobin. This transition is usually rapidly reversed upon reoxygenation, but induces progressive damage of membrane driving dehydration to the extreme to the state when HbS polymers do not dissociate as its concentration exceeds it solubility threshold. Destabilization of the membrane and decrease in deformability leads to intravascular hemolysis and to vaso-occlusive events (Ware et al., 2017). Sickle RBCs are poorly deformable (Alapan et al., 2015, 2016) and this is, in part, due to changes in hydration status of the RBC.

In α- and β-thalassemia, α- and β-hemoglobin chains are affected, respectively, which leads to an imbalance in the synthesis of globin chains and to an inability to form sufficient quantities of hemoglobin heterotetramers. The imbalanced synthesis in thalassemia leads to the formation of hemoglobin precipitates, so-called Heinz bodies. RBCs in α-thalassemia show increased hydration (Bunyaratvej et al., 1994; Chui et al., 2003), whereas the hydration of RBCs in β-thalassemia is either decreased or increased (Bunyaratvej et al., 1994; Brugnara and Mohandas, 2013). Both Heinz bodies and the altered hydration state of the RBC are known to impair RBC deformability (Clark et al., 1983; Losco et al., 2001).

#### Oxygenation of Hemoglobin

The main function of RBCs is to transport oxygen to the tissues in the human body. The oxygenation state of hemoglobin is known to influence various processes of the RBC (Gibson et al., 2000; Stefanovic et al., 2013). Several ion transporters are oxygen sensitive, such as K^+^-Cl^-^ cotransporter (KCC1) and the NKCC1 (Na-K-2Cl cotransporter) (Bogdanova et al., 2009). The function of band 3 seems, however, unaltered upon deoxygenation and oxygenation (Gibson et al., 2000). On the other hand, hemoglobin binds the cytosolic domain of band 3 and this binding is regulated by pH (Eisinger et al., 1982; Chétrite and Cassoly, 1985). Deoxygenation reduces the binding of ankyrin to band 3 and dissociation leads to more freely diffusible band 3 (Stefanovic et al., 2013). In addition, the binding between ankyrin and band 3 is also regulated by 2,3-disphosphoglycerate (2,3-DPG) (Moriyama et al., 1993). 2,3-DPG promotes the dissociation of oxygen from hemoglobin and promotes the release of oxygen from the RBC to the tissues (van Wijk and van Solinge, 2005). In the oxygenated state 2,3-DPG is not bound to hemoglobin and leads to less deformable and more fragile RBCs, while in the deoxygenated state 2,3-DPG is bound to hemoglobin and leads to an increase in RBC deformability and less fragile RBCs (Moriyama et al., 1993). The exact role of the reduced binding between ankyrin and band 3 after deoxygenation and binding of 2,3-DPG to hemoglobin is currently unknown, although it is hypothesized that a mild membrane weakening is beneficial in deoxygenated capillaries to facilitate smooth traveling of the RBC (Stefanovic et al., 2013).

#### RBC Hydration Changes Associated With Hemoglobin Defects

##### RBC dehydration in sickle cell anemia

The RBC dehydration in sickle cell anemia is caused by facilitated K^+^ loss through hyperactivated KCNN4 (also known as the Gardos Channel in RBCs) and K^+^-Cl^-^ cotransporter that is not compensated by uptake of equal amounts of Na^+^ (Clark et al., 1982; Izumo et al., 1987). Regardless of the ion transport pathway involved, RBC dehydration raises the HbS concentration, thereby affecting the equilibrium of HbS-polymerization and depolymerization in favor of the polymerized version (Eaton and Hofrichter, 1990; Brugnara, 1993). Na/K/ATPase was found to be more active in RBCs from sickle cell disease patients and can contribute to RBC dehydration (Izumo et al., 1987). Dehydration of RBCs in sickle cell disease contributes significantly to the decreased deformability since dehydration promotes the probability of polymerization of HbS by 20–40-fold (Eaton and Hofrichter, 1990) and result in prolonged polymerization of hemoglobin and thus prolonged sickling of RBCs (Brugnara, 1993). Sickled RBCs are far less deformable.

**FIGURE 6:**
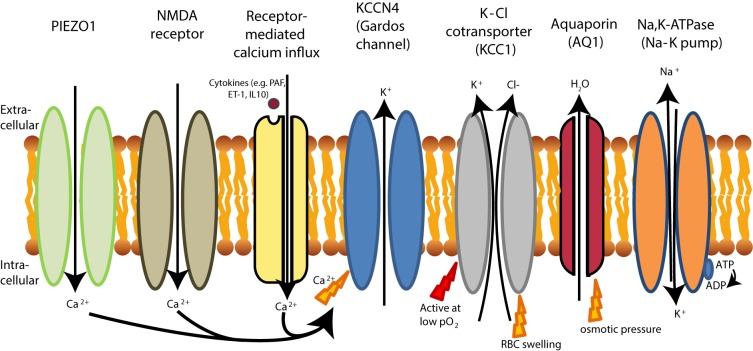
RBC dehydration in sickle cell disease. The Na-K pump (Na, K-ATPase) is more active in sickle RBCs, leading to dehydration as extrusion of 3 Na^+^ ions leads to the influx of 2 K^+^ ions. Dehydration of sickle cell is initiated by PIEZO1 activation, likely because PIEZO1 is stretch-activation after deoxygenation (Ma et al., 2012). Dehydration in sickle cell disease is also initiated by the upregulation of the NMDA receptor. Both the NMDA receptor and PIEZO1 activation result in Ca^2+^ influx that leads to KCNN4 activation (KCCN4 in RBCs is better known as the Gardos channel). Gardos channel activation in sickle cell disease is also achieved by signaling cascades which respond to increased levels of cytokines. This leads to K^+^ and water efflux by aquaporins (AQ1). In sickle cell disease, the K-Cl cotransporter is also activated at low oxygen tensions, causing efflux of K^+^ and subsequently loss of water by aquaporins (AQ1), while under normal conditions KCC is only active at normal pO_2_. Although water can be cotransported through KCC1 (Zeuthen and Macaulay, 2012), water efflux in RBCs mainly driven by aquaporins (AQ1).

Two mechanisms involved in abnormally high K^+^ loss are presented in **Figure 6**. The first mechanism of K^+^ loss involves the ubiquitously expressed K-Cl co-transporter (KCC), of which the isoforms KCC1, KCC3, and KCC4 are expressed in human RBCs (Crable et al., 2005).

KCC1, is most often studied in RBCs and referred to as KCC. KCC in RBCs are known to be regulated by intracellular pH, Mg^2+^ concentrations as well as volume and oxygenation states of hemoglobin (Adragna et al., 2004; Khan et al., 2004). Under physiological conditions, KCC activation restores cell volume after swelling, but KCC activation may also respond to the changes in the RBC redox state (Adragna et al., 2004). In healthy RBCs, KCC is oxygen sensitive but only activated at high oxygen tension (pO_2_). At low oxygen tension, KCC becomes sensitive to other stimuli (Gibson et al., 1998, 2001; Muzyamba et al., 2006). In sickle cell disease, in contrast, KCC is also activated at low oxygen tension, most likely due to increased phosphorylation of KCC in sickle RBCs (Muzyamba et al., 2006). In addition, KCC activity is intrinsically higher, even in older sickle RBCs (Bize et al., 2003).

The loss of deformability caused by the formation of HbS-polymers at low oxygen tensions (Odièvre et al., 2011) is enhanced by the KCC activation and leads to additional cell shrinkage. The clinical relevance of KCC activity is emphasized by observations in individuals with mild sickle cell disease. In these patients, an increased K-Cl cotransport activity is associated with increased likelihood of hospitalization, because of acute vaso-occlusive problems caused by less deformable dehydrated RBCs (Rees et al., 2015).

One more trigger of K^+^ leak from RBCs is the increase in the intracellular Ca^2+^ levels and the activation of Ca^2+^-sensitive K^+^ channels (KCNN4 or known as the Gardos channel in RBCs) (Gallagher, 2017). These channels driving K^+^ loss and dehydration are hyperactivated in RBCs of sickle cell disease patients secondary to the high intracellular Ca^2+^ levels (Bookchin and Lew, 1980; Bogdanova et al., 2013) (see **Figure 6**). KCNN4 is expressed in various cell types (Gallagher, 2013) and are inhibited by imidazole antimycotics, such as clotrimazole (McNaughton-Smith et al., 2008). In sickle cell disease, KCNN4 (or known as the Gardos channel in RBCs) is activated by two pathways. Firstly, KCNN4 can be activated by a signaling cascade initially triggered by several cytokines, such as PAF, interleukin-10 (IL-10) and endothelin 1 (ET-1) (Rivera, 2002) also involving activation of PKCα (Rivera et al., 1999; Wagner-Britz et al., 2013). In addition, PAF and ET-1 concentrations are increased in plasma from patients with sickle cell disease and are assumed to contribute to the adhesion of sickle RBCs to endothelium that is responsible for the vaso-occlusive events (Rivera, 2002). Also, these cytokines generate denser RBCs through RBC dehydration after an oxygenation/deoxygenation cycle (Rivera, 2002). Secondly, sickle cell disease patients have increased expression of the NMDA-receptor on their RBC membranes, causing pathological influx of Ca^2+^ after stimulation with receptor agonists (such as glycine and homocysteic acid) (Hänggi et al., 2014) which could contribute to dehydration mediated by KCNN4. Currently, a clinical trial using memantine as a NMDA-receptor antagonist is being tested in sickle cell disease patients (Bogdanova et al., 2017). In addition, the KCNN4 (or Gardos channel) blocker senicapoc (ICA-17043) has recently been tested in sickle cell disease. Senicapoc increased hemoglobin concentrations and hematocrit, and improved RBC hydration. Despite these encouraging findings, this study has been terminated prematurely because senicapoc did not meet its primary efficacy endpoint, defined as a decrease in painful crises (Ataga et al., 2011). From the clinical results obtained with senicapoc it can be hypothesized that the RBC dehydration and changes in RBC hydration after treatment with senicapoc are not causally related to the vaso-occlusive events which are often observed in sickle cell anemia.

Another pathway that increases intracellular Ca^2+^ levels in sickle cell disease is the mechanosensitive PIEZO1 channel (also described at section “Primary Changes of RBC Hydration”). PIEZO1 channels are widely expressed in vertebrates across various cell types and in RBC these channels are responsible for regulation of volume homeostasis upon mechanical signals (Zarychanski et al., 2012; Ge et al., 2015). Sickle RBCs show increased permeability for Ca^2+^ and Mg^2+^ upon deoxygenation. This increased permeability results in an increased deoxygenation-induced cation conductance in sickle cell disease, which can be entirely blocked by GsMTx4 (Ma et al., 2012; Cahalan et al., 2015). The gating modifier GsMTx4 blocks the mechanically sensitive part of PIEZO1 that supports a closed state of this ion channel (Bae et al., 2011), preventing Ca^2+^ influx (Jacques-Fricke et al., 2006).

##### RBC hydration changes in thalassemia

Three increasingly severe phenotypes can be distinguished in β-thalassemia, i.e., β-thalassemia minor, intermedia and major (Higgs et al., 2012). Intracellular concentrations of Na^+^ in RBCs from patients with β-thalassemia minor and β-thalassemia major are comparable with those in RBCs from healthy adults, but K^+^ levels are slightly increased (Cividalli et al., 1971). Upon *in vitro* incubation at 37°C, RBCs from patients with β-thalassemia major show increased leakage of K^+^ and elevation of intracellular Na^+^ levels when compared with healthy RBCs. In RBCs from β-thalassemia minor patients, leakage of K^+^ and intracellular Na^+^ levels are comparable to the healthy RBCs after *in vitro* incubation at 37°C (Cividalli et al., 1971).

Leakage of K^+^ from RBCs in thalassemia is possibly linked to the precipitation of excess α- or β-globin chains inside the RBCs, which is a characteristic feature of this disease. The precipitated globin chains in RBCs can lead to oxidative stress and can affect transport of Na^+^ and K^+^ in thalassemia RBCs (Olivieri et al., 1994). In both β-thalassemia and α-thalassemia, RBCs show increased efflux of K^+^ (Olivieri et al., 1994). Moreover, the amount of hemoglobin aggregates in RBCs is correlated with K^+^ efflux (Nathan and Gunn, 1966). The presence of these aggregates reduces RBC deformability (Lubin and Desforges, 1972; Hasegawa et al., 1993) thereby revealing correlation between K^+^ fluxes, Heinz body formation, and reduced RBC deformability. The precipitation of globin chains and leakage of K^+^ from RBCs in thalassemia is also linked, or may even be augmented, by overload of Ca^2+^ (Shalev et al., 1984; Bookchin et al., 1988).

Another important factor in K^+^ loss from RBCs in β-thalassemia involves the K^+^-Cl^-^ cotransporter, which can be affected by oxidative stress (Olivieri et al., 1994). This K^+^ loss by the K^+^-Cl^-^ cotransporter is inhibited by increased intracellular concentrations of Mg^2+^ (De Franceschi et al., 1998; Adragna et al., 2004). In a trial with β-thalassemia intermedia patients, dietary Mg^2+^ supplementation did lead to increased intracellular concentrations of Mg^2+^ and to a reduction in the activities for the K^+^-Cl^-^ cotransporter and Na-K pump (Na/K/ATPase). However, Mg^2+^ did not influence the activities of NKCC1 (also known as the Na-K-Cl cotransporter). Although dietary Mg^2+^ supplementation in β-thalassemia patients does not influence the hemoglobin concentration in blood, it does lead to a significant decrease in the absolute reticulocyte number. This is possibly due to improved RBC survival time (De Franceschi et al., 1998).

Little research has been performed on KCNN4 (or also known as the Gardos channel in RBCs) in thalassemia. An exception is the work on a mouse model with a homozygous deletion in the β-globin chain (de Franceschi et al., 1996). These mice were treated with clotrimazole, an antifungal imidazole derivate with KCNN4 blocking properties. During treatment with clotrimazole, hemoglobin concentrations in blood remained constant. In addition, it caused a decrease in MCHC whereas hematocrit and intracellular K^+^ levels increased indicating that hydration state of the RBC was restored. Combination of clotrimazole with erythropoietin administration did increase hemoglobin levels in these mice to a higher extent than erythropoietin alone, possibly by promoting proliferation and differentiation during erythropoiesis (de Franceschi et al., 1996).

### RBC Vesiculation

**FIGURE 7:**
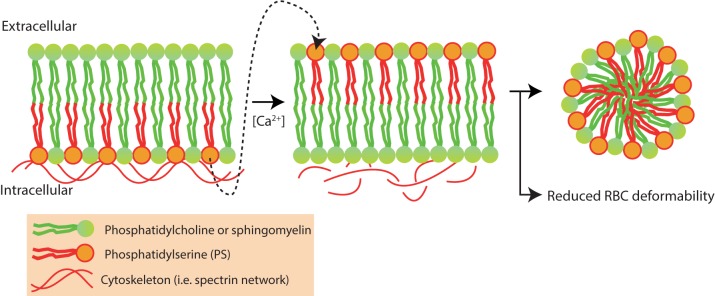
Red blood cell vesiculation is driven by increased concentrations of intracellular Ca^2+^. Phosphatidylserine (PS) is normally presented in the inner-leaflet of the RBC. Elevated intracellular Ca^2+^ levels activate scramblase and inhibit flippase, leading to phosphatidylserine (PS) externalization and redistribution of membrane and cytoskeletal proteins. PS externalization and protein redistribution leads to vesicle formation and loss of RBC deformability (Alaarg et al., 2013).

Intracellular levels of Ca^2+^ tightly regulate RBC vesiculation and elevated levels of intracellular Ca^2+^ are known to induce PS exposure and RBC vesiculation (Bevers et al., 1992; Nguyen et al., 2011; Fens et al., 2012; Alaarg et al., 2013) (**Figure 7**).

The RBC-vesiculation is a physiological process and leads to reduced RBC deformability. During its 120-day life span, RBC lose membrane surface and hemoglobin content through vesiculation (Werre et al., 2004; Ciana et al., 2017) a process in particular prominent in the youngest RBCs (Greenwalt and Dumaswala, 1988). As membrane is shed, this leads to a decreased surface-to-volume ratio in the RBC. During this RBC aging, hemoglobin-free vesicles are shed which leads to RBC dehydration and older RBCs with increased MCHC (Bosch et al., 1994; Bosman, 2013). Altogether, this leads to a reduction in membrane elasticity and RBC deformability (Linderkamp et al., 1993; Bosch et al., 1994). This implies that there is an important role for vesiculation in RBC deformability.

While RBC-vesiculation is considered a physiological process, RBC vesiculation is increased in various forms of hereditary hemolytic anemia, such as HS and sickle cell disease (Perrotta et al., 2008; Alaarg et al., 2013). In HS, differences in deformability between spectrin/ankyrin-deficient and band 3-deficient RBCs were observed after splenectomy. While splenectomy prevented premature removal of young RBCs from the circulation in both groups, the loss of deformability during RBC-aging was delayed after splenectomy in spectrin/ankyrin-deficient RBCs. This contrasts with band 3-deficient RBCs, where splenectomy did not lead to a delay in the deformability decrease during RBC-aging. This could be explained by the fact that spectrin/ankyrin-deficient RBCs are more prone to shed band 3-containing vesicles. Clustered band 3 is known to induce binding of autologous IgG, which facilitates removal of RBC by macrophages (Kay et al., 1983; Kay, 1984; Willekens et al., 2008). Shedding of band 3-containing vesicles from spectrin/ankyrin-deficient RBCs, therefore, would avoid IgG-opsonisation and clearance of the RBC (Reliene et al., 2002).

Red blood cell vesiculation is suggested as a mechanism to protect the cell from removal from the circulation. By vesiculation, the RBC can shed “eat-me” signals such as PS and specific band 3 cleavage products that react with senescent antigens (Willekens et al., 2008). By shedding these “eat-me” signals, the RBC may escape clearance until the reduction in RBC deformability causes trapping in the spleen. In addition, the lipid bilayer of the RBC is a complex system with various microdomains. These microdomains of the RBC, or so called ‘lipid rafts’ are specific membrane parts with high concentrations of cholesterol, sphingomyelin and gangliosides (Pike, 2003). These specific lipid rafts have a high abundance of the membrane proteins stomatin, flotillin-1, flottilin-2 (Salzer and Prohaska, 2001) and band 3 (Cai et al., 2012). RBC vesicles shed during storage are enriched in the lipid raft marker stomatin (Salzer et al., 2008) and indicates that specific lipid domains are shed during RBC vesiculation (Leonard et al., 2017).

The relationship between vesiculation and deformability is nicely illustrated by an experiment where chlorpromazine is added to RBCs to stabilize the RBC membrane. During overnight incubation with chlorpromazine at 37°C in a glucose-free buffer, vesiculation is inhibited and RBC deformability is maintained. Without chlorpromazine strong vesiculation of RBCs under these conditions occur as PS is exposed on the RBC surface. The activity of chlorpromazine may be explained by the amphiphilic properties of the molecule directly affecting the RBC membrane (Bütikofer et al., 1989).

High-throughput screening with chemical compound libraries also revealed that RBC-vesiculation can be driven by drugs and chemical compounds. For example, vesiculation of RBCs is driven by certain kinase pathways, including Jak-STAT and protein kinase B. Increased RBC vesiculation is also observed after addition of paclitaxel to blood, probably through its formulation excipient containing Cremophor. Not only RBC vesiculation is influenced by the vehicle Cremophor, but the compound also increases whole blood viscosity and transforms RBC to stomatocytes (Vader et al., 2013).

### Adaptive Responses

Exercise leads to increased heart rate as a response to provide tissues with sufficient amount of oxygen. Exercise is accompanied with several stress factors that may affect RBC deformability, such as shear stress, hyperthermia, and glucose consumption (Carlson and Mawdsley, 1986; Szygula, 1990; Smith, 1995; Mairbäurl, 2013). These stress factors can lead to mechanical rupture, stimulated erythropoiesis and can decrease the average RBC age in athletes (Mairbäurl, 2013).

In a study with 24 trained cyclists, RBC deformability was decreased directly after exercise (Oostenbrug et al., 1997). The biology beyond the decrease in RBC deformability is unknown, although increased blood flow may be involved. The change in deformability after exercise can be the results of cytoskeletal changes, such as membrane loss. In addition, decreased levels of haptoglobin and increased levels of bilirubin were observed after marathon races (Jordan et al., 1998; Simpson et al., 2006). On the other hand, RBC deformability seems to be generally increased in well-trained athletes (Mairbäurl, 2013). A study comparing RBC membrane fluidity with the RBC membrane composition in controls and runners observed increased RBC membrane fluidity in endurance runners and sprinters. Although the intake of nutrients was comparable between the running groups and the control group, endurance running was accompanied with reduced concentrations of saturated fatty acids in RBC membranes (Kamada et al., 1993). These results indicate that the RBC can adapt upon exposure to exercise and that these adapted RBCs facilitate exercise and proper delivery of oxygen to the tissues. Interestingly, Smith et al. (1999) observed a higher maximal RBC deformability in elite cyclists when compared with sedentary healthy controls. Also, RBC populations with lower RBC densities were observed in the elite cyclists, which could indicate increased RBC turnover (Smith et al., 1999). A possible explanation for the increased RBC deformability in elite cyclist could be that extreme exercise leads to membrane loss and early RBC uptake, which is likely to be compensated with relatively young and good deformable RBCs. Whether increased RBC deformability would lead to better sports performances is unknown.

Autologous erythropoietin production is stimulated upon exposure to high altitudes. Acute exposure to high altitude, however, does not affect RBC deformability and RBC rheology (Reinhart et al., 1991). The effects of chronic exposure to high altitude seems largely unexplored. RBC deformability related processes were investigated in RBC concentrates obtained from Tibetans living at high altitude and from Tibetans at living at lowland. RBC viscosity was increased, and RBCs were more osmotic fragile in Tibetians living at low altitude when compared to RBC concentrates obtained from their lowland residents (Zhong et al., 2015). The increased viscosity can, however, be affected by increased hemoglobin levels and the decreased osmotic fragility can be affected by the decreased MCV values in Tibetans living at high altitude. The effects of exercise under hypoxia on RBC properties were investigated by Mao et al. (2011). Exercise under hypoxic conditions was found to decrease KCNN4 (or Gardos-channel) modulated deformability and KCNN4 modulated volume and down-regulated the senescence markers CD47 and CD147 (Mao et al., 2011). These results indicate RBC dysfunction after exercise under hypoxia. The dysfunction of RBCs under hypoxic conditions in combination with the erythropoietin-driven increase in RBC production leads to RBCs with relative low densities (Samaja et al., 1993), possibly caused by both increased RBC turnover and increased RBC production.

## Summary

Deformability is an important parameter that regulates RBC rheology, its longevity, and efficacy of O_2_ transport. Altered deformability is a characteristic feature of multiple forms of hereditary hemolytic anemias and is likely related to the severity of the disease. Factors regulating deformability at the cellular level are dehydration, membrane protein phosphorylation, cytoskeletal integrity, metabolism and the integrity of hemoglobin. Interaction of these factors makes RBCs more or less deformable. Measuring RBC deformability in research and diagnostic laboratories can be challenging as there are many techniques available with all their specific advantages and disadvantages.

Measuring RBC deformability is important from both a diagnostic and research point of view. RBC deformability (i) could provide information about the patient’s disease and clinical severity, and (ii) could be a target for pharmacological intervention or predict the toxicity of drugs for patients with hemolytic anemias.

Reduced RBC deformability leads to an inability of the RBC to pass the splenic circulation and leads to premature removal of RBCs from the blood. Altered RBC deformability can be attributed to primary and secondary changes of RBC deformability. Primary changes of RBC deformability are directly related to the disease, such as the membrane weakening in HS or the formation of poorly deformable sickle cells in sickle cell disease. Secondary changes of RBC deformability, such as altered ion fluxes, aberrant membrane protein phosphorylation or RBC vesiculation, are not directly related to the cause of disease. Thus, both primary and secondary causes of RBC deformability can contribute to premature uptake of RBCs in the spleen. In this review and in **Table 2**, we have summarized the current knowledge on primary and secondary mechanisms of RBC deformability in sickle cell anemia, thalassemia, HS, hereditary xerocytosis, and a number of metabolic disorders of the RBC. We have addressed and discussed the effects of ion regulation, ion channels and RBC (de)hydration on RBC deformability. In addition, we discuss the role and current knowledge of the adaptive responses on RBC deformability, intracellular energy-sensing molecules, membrane protein phosphorylation, hemoglobin deoxygenation and RBC vesiculation on RBC deformability. Knowledge about these processes in the RBC will lead to better understanding of the secondary processes involved in premature removal, and could lead to the discovery of new targets for pharmacological treatment. Furthermore, we postulate that a number of currently undiagnosed, patients with hereditary hemolytic anemia may have (genetic) defects in the here discussed secondary pathways or secondary processes that regulate RBC deformability. This review helps to understand the molecular mechanisms that maintain RBC deformability in healthy and diseased individuals, and enumerates the molecular mechanisms that are altered in RBC disorders leading to hereditary hemolytic anemia.

**Table 2:** Summary of primary and secondary changes that lead to reduced RBC deformability in hereditary hemolytic anemia.

Anemia group	Disorder	Affected protein	Secondary changes	Deformability
Hemoglobinopathies	Sickle cell anemia	β globin	High intracellular free Ca^2+^ dehydration oxidation, hemolysis, NO scavenging, KCNN4 (Gardos channel) activation, different KCC activity	↓
	Thalassemia	Deficiency in α or β globin chain	High intracellular free Ca^2+^	↓
Metabolic disorders	Pyruvate kinase deficiency (PKD)	PK	Changes in phosphorylate, low ATP, low cAMP, AMPK activation, intracellular free Ca^2+^ overload	↓, but dependent on technique
	Hexokinase (HK) deficiency	HK		↓, but dependent on technique
	Glucose-6-phosphate-dehydrogenase	G6PD	AMPK activation when in hemolytic crisis	Not affected, but ↓ when in crisis
Structural disorders	Hereditary Spherocytosis	Band 3, ankyrin, SPTA1, SPTB, RhAG, protein 4.1, protein 4.2	Increased MCHC, RBC vesiculation, low intracellular K^+^	↓
	Hereditary elliptocytosis	SPTA1, SPTB or protein 4.1	Formation of elliptocytes	↓
Channelopathies	Hereditary xerocytosis	PIEZO1	Low K^+^, RBC dehydration	Slightly decreased, mainly dehydrated Splenectomy contra-indicated
	Gardos channelopathy	Gardos channel	Low intracellular K^+^, RBC dehydration, increased Ca^2+^	Slightly decreased, mainly dehydrated


*Reduced RBC deformability is noted in the table with ↓.*

## Author Contributions

RvW, RS, and WvS encouraged RH to investigate the properties of RBC deformability. RH and RvW wrote the manuscript with support from RS, AB, LK, and WvS.

## Conflict of Interest Statement

The authors declare that the research was conducted in the absence of any commercial or financial relationships that could be construed as a potential conflict of interest.

## Abbreviations

5-FU: 5-fluorouracil
AC: adenyl cyclase
AMPK: AMP-activated protein kinase
ATP: adenosine triphosphate
Ca^2+^: calcium
cAMP: cyclic adenosine monophosphate
cGMP: cyclic guanosine monophosphate
Cl^-^: chloride
DNA: deoxyribonucleic acid
ET-1: endothelin-1
GAPDH: glyceraldehyde 3-phosphate dehydrogenase
HbS: hemoglobin S
HK: hexokinase
HS: hereditary spherocytosis
IL-10: interleukine-10
K^+^: potassium
KCC: = K^+^-Cl^-^ cotransporter
KCNN4: calcium-activated potassium channel or Gardos channel
MCHC: mean corpuscular hemoglobin concentration
Mg^2+^: magnesium
Na^+^: sodium
PAF: platelet-activating factor
PDE: phosphodiesterase
PK: pyruvate kinase
PKA: protein kinase A
PKCα: protein kinase C alpha
PS: phosphatidylserine
RBCs: red blood cells
RNA: ribonucleic acid
